# Do patients with unilateral macular neovascularization type 3 need AREDS supplements to slow the progression to advanced age-related macular degeneration?

**DOI:** 10.1038/s41433-022-02249-2

**Published:** 2022-09-29

**Authors:** Bilal Haj Najeeb, Ursula Schmidt-Erfurth

**Affiliations:** Department of Ophthalmology, Medical University of Vienna, Vienna, Austria

**Keywords:** Macular degeneration, Outcomes research

## Abstract

Given the wide spectrum of unique characteristics of macular neovascularization type 3 (MNV3) compared with types 1 and 2, we suggest regrading the colour photography assessment of the AREDS study to verify the impact of AREDS supplements on eyes with MNV3.

The Age-Related Eye Disease Study (AREDS), which was launched in 1992, demonstrated that oral supplements of antioxidant vitamins and minerals have beneficial effects in patients who develop advanced age-related macular degeneration (AMD), in those with at least intermediate AMD, defined as bilateral large drusen with or without pigment changes [1]. An individual eye was classified as having progressed to advanced AMD when it develops a vision-threatening lesion. These lesions included geographic atrophy involving the fovea and macular neovascularization (MNV).

In recent years, MNV type 3 (MNV3), also known as retinal angiomatous proliferation, has been shown to demonstrate apparent clinical and pathomorphological features which clearly distinguish it from the other two types (MNV1 and 2). The most important characteristics are (1) pathological: the neovascularization has a dual (retinal and choroidal) origin [2], (2) laterality: in almost all cases it is bilateral [3–5] with the fellow eye usually becoming involved within several months, if not simultaneously [3, 6–8], (3) genetical: MNV3 is less associated with relevant AMD genotypes and complement factors such as CFH Y402 [9], (4) demographical: patients with MNV3 are older than those with other types [9, 10], even though there is no concurrent or previous MNV1 or 2 lesions in eyes with newly diagnosed MNV3 [8, 11, 12], (5) topographical: contrary to other types, MNV3 is mainly distributed in the temporal half of the macula [12], (6) morphological: (a) newly diagnosed cases mainly present with intraretinal haemorrhage and surrounding dense hard exudates, but not with subretinal haemorrhage [10, 13], (b) subretinal drusenoid deposits (reticular pseudodrusen) are predominantly seen in MNV3, whereas drusen, which were the main inclusion criterion in the AREDS, occur much less often [14, 15], (7) environmental: hypertension is more commonly seen in patients with MNV3 [9], (8) choroidal: eyes with MNV3 have significant choroidal thinning and reduced perfusion [16, 17], (9) unique phenotypes: such as the cilioretinal variant, when the neovascularization arises from a cilioretinal artery, or the multifocal variant, when there is more than one MNV3 lesion, or the development of retinal-choroidal anastomosis (RCA) [8, 11, 18, 19].

This wide spectrum of characteristic features confined to the third type of MNV raises the vital question as to whether the AREDS formula can realistically reduce the incidence of advanced AMD in the fellow eyes of patients with newly diagnosed unilateral MNV3. Unfortunately, existing AREDS reports cannot provide an answer, as all patients who developed advanced AMD were pooled together for data analysis without a predetermination of their type of MNV and anyway MNV3 was only first reported in 2001 [2].

The possibility that supplements do not significantly decrease the incidence of advanced AMD in patients in the MNV3 group has critical clinical and economic implications for AMD research and management. There may be no need for a lifelong intake of AREDS supplements by patients with unilateral MNV3, who comprise a substantial proportion up to 35% of all patients with MNV [20]. Indeed, caution is especially important due to the very advanced age of these patients. Moreover, all robust outcomes of the AREDS reports on MNV, such as the impact on public health, the association with genetic variants, hyperopia, drusen, lens opacities, education level, gender, body mass index, and ethnic group (AREDS reports 3, 11, 14), should be adjusted for two subgroups: MNV3 and no MNV3. Accordingly, the specific effect of AREDS supplements on the MNV1/2 groups could also be adjusted.

Optical coherence topography or indocyanine green angiography is usually necessary to confirm an MNV3 diagnosis, but neither was available in the AREDS. However, in our third report of the RAP study, “Discoloration of the macular region in patients with macular neovascularization type 3” [13], we were able to diagnose MNV3 using only colour photography (CP), which was the standard examination in the AREDS. In our report, using multimodal imaging, we found that specifically *intraretinal* haemorrhage, which was not verified in the AREDS reports [21], occurs in the vast majority of cases, and exclusively in eyes with MNV3. The intraretinal haemorrhage overlying the lesion presents as a solitary profuse splinter shape in the inner retinal layers, but further away from the lesion the haemorrhage extends into the intraretinal cysts giving the lesion a pattern of numerous punctate/semi punctate bleedings. This unique pattern of distribution of the haemorrhage on CP helps to differentiate the intraretinal from the subretinal location, where haemorrhages typically occur in newly diagnosed MNV1/2, but not in MNV3 [13]. A very high percentage (90%) of intraretinal haemorrhage was also found in patients with MNV3 in the CATT study [10]. Thus, intraretinal haemorrhage is pathognomonic for MNV3 and can be a used as a valid biomarker to diagnose MNV3 on CF, i.e., clinically. Moreover, in the same report, dense exudate on CP was found in about two-thirds of MNV3 eyes, significantly more than seen in MNV1 or 2 [13].

Furthermore, in our fifth RAP report, “rediscovering MNV3, multimodal imaging of fellow eyes over 24 months [19]”, we showed that MNV3 eyes had a pathognomonic RCA in 40% of scarred lesions, whereas other MNV types did not present with RCA. It manifests on CF as an enlarged tortuous vessel (usually arteriole) which suddenly disappears into the deeper scarred tissue in the perifoveal area. Thus, MNV3 has a second exclusive feature on CF which helps to diagnose longstanding fibrotic MNV3 cases accounting for 37% of all MNV3 cases according to the natural course of this disease [2].

Notably, as MNV3 is a bilateral disease, without concurrent MNV1 or 2 lesions even in its multifocal phenotype [3–5, 8, 12], the presence of these distinguishing characteristics (intraretinal haemorrhage, RCA) in at least one eye is enough to identify type 3 in the partner eye when exudative changes develop. This also facilitates recognition of MNV3 cases in AREDS eyes at the earliest timepoint, which is of particular importance in timely treatment of MNV3.

Finally, the trigger for the AREDS study was public health concern about the wide-spread intake of unproven, high-dose antioxidants. Plausibly, the effect of AREDS supplements on patients with MNV3 should also be evaluated in light of today´s knowledge. Therefore, we recommend regrading CP of AREDS eyes to clearly identify the particular effect of AREDS supplements on MNV3 eyes.

## References

[1] Age-Related Eye Disease Study Research Group. A randomized, placebo-controlled, clinical trial of high-dose supplementation with vitamins C and E, beta carotene, and zinc for age-related macular degeneration and vision loss. Arch Ophthalmol. 2001;119:1417. 10.1001/archopht.119.10.1417. 10.1001/archopht.119.10.1417 PMC1462955 11594942

[2] Yannuzzi LA, Negrão S, Lida T, Carvalho C, Rodriguez-Coleman H, Slakter J (2001). Retinal angiomatous proliferation in age-related macular degeneration. Retina..

[3] Gross NE, Aizman A, Brucker A, Klancnik JM, Yannuzzi LA (2005). Nature and risk of neovascularization in the fellow eye of patients with unilateral retinal angiomatous proliferation. Retina.

[4] Sawa M, Ueno C, Gomi F, Nishida K (2014). Incidence and characteristics of neovascularization in fellow eyes of Japanese patients with unilateral retinal angiomatous proliferation. Retina.

[5] Campa C, Harding SP, Pearce IA, Beare NAV, Briggs MC, Heimann H. Incidence of neovascularization in the fellow eye of patients with unilateral retinal angiomatous proliferation. Eye. 2010. 10.1038/eye.2010.88. 10.1038/eye.2010.88 20539314

[6] Wong TY, Lanzetta P, Bandello F, Eldem B, Navarro R, Loevestam-Adrian M (2020). Current concepts and modalities for monitoring the fellow eye in neovascular age-related macular degeneration. Retina.

[7] Kim JH, Kim JW, Kim CG, Lee DW (2020). Influence of fellow-eye examination interval on visual acuity at fellow-eye neovascularization in unilateral type 3 neovascularization. Retina.

[8] Haj Najeeb B, Deak GG, Sacu S, Schmidt-Erfurth U, Gerendas BS. The RAP study, report 4: morphological and topographical characteristics of multifocal macular neovascularization type 3. Graefe’s Arch Clin Exp Ophthalmol. 2021. 10.1007/s00417-021-05332-8. 10.1007/s00417-021-05332-8 PMC8763817 34436646

[9] Caramoy A, Ristau T, Lechanteur YT, Ersoy L, Mueller S, Gelisken F (2014). Environmental and genetic risk factors for retinal angiomatous proliferation. Acta Ophthalmol.

[10] Daniel E, Shaffer J, Ying G, Grunwald JE, Martin DF, Jaffe GJ (2016). Outcomes in eyes with retinal angiomatous proliferation in the comparison of age-related macular degeneration treatments trials (CATT). Ophthalmology..

[11] Haj Najeeb B, Deak GG, Schmidt-Erfurth UM, Gerendas BS (2021). RAP study, report 1: novel subtype of macular neovascularisation type III, cilioretinal MNV3. Br J Ophthalmol.

[12] Haj Najeeb B, Deak G, Schmidt-Erfurth U, Gerendas BS (2020). THE RAP STUDY, REPORT TWO: the regional distribution of macular neovascularization type 3, a novel insight into its etiology. Retina.

[13] Haj Najeeb B, Deak GG, Schmidt‐Erfurth U, Gerendas BS. The RAP study, report 3: Discoloration of the macular region in patients with macular neovascularization type 3. Acta Ophthalmol. 2021:aos.14866. 10.1111/aos.14866. 10.1111/aos.14866 PMC9292611 33821577

[14] Marsiglia M, Boddu S, Chen CY, Jung JJ, Mrejen S, Gallego-Pinazo R (2015). Correlation between neovascular lesion type and clinical characteristics of nonneovascular fellow eyes in patients with unilateral, neovascular age-related macular degeneration. Retina.

[15] Marques JP, Laíns I, Costa MÂ, Pires I, da luz Cachulo M, Figueira J (2015). Retinal angiomatous proliferation. Retina.

[16] Borrelli E, Souied EH, Freund KB, Querques G, Miere A, Gal-Or O (2018). Reduced choriocapillaris flow in eyes with type 3 neovascularization and age-related macular degeneration. Retina.

[17] Koizumi H, Iida T, Saito M, Nagayama D, Maruko I (2008). Choroidal circulatory disturbances associated with retinal angiomatous proliferation on indocyanine green angiography. Graefe’s Arch Clin Exp Ophthalmol.

[18] Kim JH, Chang YS, Kim JW, Kim CG, Lee DW (2020). Characteristics of type 3 neovascularization lesions. Retina.

[19] Haj Najeeb B, Deak GG, Mylonas G, Sacu S, Gerendas BS, Schmidt-Erfurth U (2022). THE RAP STUDY, REPORT 5: REDISCOVERING MACULAR NEOVASCULARIZATION TYPE 3: multimodal imaging of fellow eyes over 24 months. Retina.

[20] Jung JJ, Chen CY, Mrejen S, Gallego-Pinazo R, Xu L, Marsiglia M (2014). The incidence of neovascular subtypes in newly diagnosed neovascular age-related macular degeneration. Am J Ophthalmol.

[21] The age-related eye disease study system for classifying age-related macular degeneration from stereoscopic color fundus photographs: the age-related eye disease study report number 6. Am J Ophthalmol. 2001;132:668-81. 10.1016/S0002-9394(01)01218-1. 10.1016/s0002-9394(01)01218-1 11704028

